# 1062. Analysis of Resistance to Oral Standard of Care Antibiotics for Urinary Tract Infections Caused By *Escherichia coli* and *Staphylococcus saprophyticus* Collected Worldwide between 2019-2020

**DOI:** 10.1093/ofid/ofab466.1256

**Published:** 2021-12-04

**Authors:** S J Ryan Arends, Deborah Butler, Nicole Scangarella-Oman, Lindsey Paustian, Jennifer M Streit, Rodrigo E Mendes

**Affiliations:** 1 JMI Laboratories, North Liberty, Iowa; 2 GSK, Collegeville, Pennsylvania; 3 GlaxoSmithKline Pharmaceuticals, Collegeville, Pennsylvania

## Abstract

**Background:**

Gepotidacin (GSK2140944) is a novel triazaacenaphthylene bacterial type II topoisomerase inhibitor under development for the treatment of gonorrhea and uncomplicated urinary tract infections (UTI). This study reports on the *in vitro* activity of gepotidacin and other oral antibiotics when tested against contemporary *Escherichia coli* and *Staphylococcus saprophyticus* clinical isolates collected from patients with UTIs for a gepotidacin uUTI global surveillance study as a part of the SENTRY Antimicrobial Surveillance Program.

**Methods:**

A total of 3,562 *E. coli* and 344 *S. saprophyticus* isolates were collected between 2019 and 2020 from 92 medical centers located in 25 countries. Most isolates (68%) tested were cultured from urine specimens collected from patients seen in ambulatory, emergency, family practice, and outpatient medical services. Bacterial identifications were confirmed by MALDI-TOF. Isolates were tested for susceptibility by CLSI methods at a central laboratory (JMI Laboratories). MIC results for oral antibiotics licensed for the treatment of uUTI and drug-resistant subsets were interpreted per CLSI guidelines.

**Results:**

Gepotidacin (MIC_50/90_, 2/2 mg/L) displayed good activity against 3,562 *E. coli* isolates, with 98.0% of all observed gepotidacin MICs ≤4 mg/L (Table). Susceptibility (S) rates for the other oral agents tested against these isolates were: amoxicillin-clavulanate (79.6% S), ampicillin (45.6% S), ciprofloxacin (72.5%S), fosfomycin (99.0% S), mecillinam (94.1%S), nitrofurantoin (97.3% S), and trimethoprim-sulfamethoxazole (68.2% S). When tested against the drug-resistant subsets, gepotidacin maintained similar MIC_50/90_ values (2/4 mg/L), except against isolates resistant to fosfomycin (2/8 mg/L). Against *S. saprophyticus* isolates, gepotidacin (MIC_50/90_, 0.06/0.12 mg/L) inhibited all isolates at ≤0.25 mg/L. Most oral agents showed S results of >97% against *S. saprophyticus* isolates, except for penicillin (3.5%S).

**Conclusion:**

Gepotidacin demonstrated potent *in vitro* activity against contemporary *E. coli* and *S. saprophyticus* urine isolates. This activity was largely unaffected among isolates demonstrating drug-resistance to other oral standard of care antibiotics.

Table

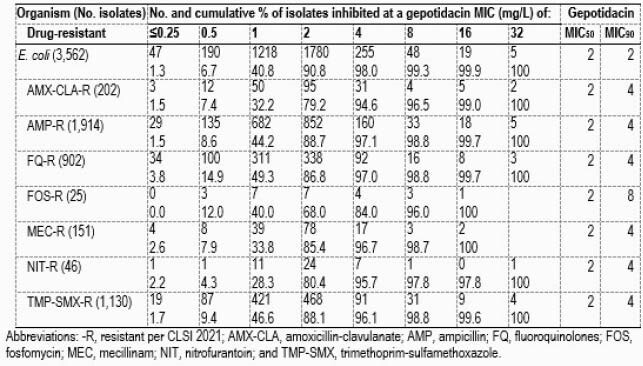

**Disclosures:**

**S J Ryan Arends, PhD**, **AbbVie (formerly Allergan**) (Research Grant or Support)**GlaxoSmithKline, LLC** (Research Grant or Support)**Melinta Therapeutics, LLC** (Research Grant or Support)**Nabriva Therapeutics** (Research Grant or Support)**Spero Therapeutics** (Research Grant or Support) **Deborah Butler, n/a**, **GlaxoSmithKline, LLC** (Employee) **Nicole Scangarella-Oman, MS**, **GlaxoSmithKline, LLC** (Employee) **Lindsey Paustian, BS (ASCP**), **GlaxoSmithKline, LLC** (Research Grant or Support) **Jennifer M. Streit, BS**, **GlaxoSmithKline, LLC** (Research Grant or Support)**Melinta Therapeutics, LLC** (Research Grant or Support)**Shionogi** (Research Grant or Support)**Spero Therapeutics** (Research Grant or Support) **Rodrigo E. Mendes, PhD**, **AbbVie** (Research Grant or Support)**AbbVie (formerly Allergan**) (Research Grant or Support)**Cipla Therapeutics** (Research Grant or Support)**Cipla USA Inc.** (Research Grant or Support)**ContraFect Corporation** (Research Grant or Support)**GlaxoSmithKline, LLC** (Research Grant or Support)**Melinta Therapeutics, Inc.** (Research Grant or Support)**Melinta Therapeutics, LLC** (Research Grant or Support)**Nabriva Therapeutics** (Research Grant or Support)**Pfizer, Inc.** (Research Grant or Support)**Shionogi** (Research Grant or Support)**Spero Therapeutics** (Research Grant or Support)

